# A Screening Study for the Development of Simvastatin-Doxorubicin Liposomes, a Co-Formulation with Future Perspectives in Colon Cancer Therapy

**DOI:** 10.3390/pharmaceutics13101526

**Published:** 2021-09-22

**Authors:** Cristina Ioana Barbălată, Alina Silvia Porfire, Alina Sesarman, Valentin-Florian Rauca, Manuela Banciu, Dana Muntean, Rareș Știufiuc, Alin Moldovan, Cristian Moldovan, Ioan Tomuță

**Affiliations:** 1 Pharmaceutical Technology and Biopharmaceutics, Faculty of Pharmacy, Iuliu Hațieganu University of Medicine and Pharmacy, 41 Victor Babes Street, 400012 Cluj-Napoca, Romania; Barbalata.Cristina@umfcluj.ro (C.I.B.); Dana.Muntean@umfcluj.ro (D.M.); tomutaioan@umfcluj.ro (I.T.); 2 Department of Molecular Biology and Biotechnology, Centre for Systems Biology, Biodiversity and Bioresources (3B), Faculty of Biology and Geology, Babes-Bolyai University, 5-7 Clinicilor Street, 400006 Cluj-Napoca, Romania; sesarman@gmail.com (A.S.); valirauca@yahoo.co.uk (V.-F.R.); manuela.banciu@ubbcluj.ro (M.B.); 3 Molecular Biology Center, Institute for Interdisciplinary Research in Bio-Nano-Sciences of Babes-Bolyai University, 42 Treboniu Laurian Street, 400271 Cluj-Napoca, Romania; 4 MedFuture Research Center for Advanced Medicine, Iuliu Hatieganu University of Medicine and Pharmacy, 4-6 Louis Pasteur Street, 400337 Cluj-Napoca, Romania; Rares.Stiufiuc@umfcluj.ro (R.Ș.); Alin.Moldovan@umfcluj.ro (A.M.); Moldovan.cristian1994@gmail.com (C.M.)

**Keywords:** screening, QbD, Doxorubicin, Simvastatin, liposomes, colon cancer

## Abstract

An increasing number of studies published so far have evidenced the benefits of Simvastatin (SIM) and Doxorubicin (DOX) co-treatment in colorectal cancer. In view of this, the current study aimed to investigate the pharmaceutical development of liposomes co-encapsulating SIM and DOX, by implementing the Quality by Design (QbD) concept, as a means to enhance the antiproliferative effect of the co-formulation on C26 murine colon cancer cells co-cultured with macrophages. It is known that the quality profile of liposomes is dependent on the critical quality attributes (CQAs) of liposomes (drug entrapped concentration, encapsulation efficiency, size, zeta potential, and drug release profile), which are, in turn, directly influenced by various formulation factors and processing parameters. By using the design of experiments, it was possible to outline the increased variability of CQAs in relation to formulation factors and identify by means of statistical analysis the material attributes that are critical (phospholipids, DOX and SIM concentration) for the quality of the co-formulation. The in vitro studies performed on a murine colon cancer cell line highlighted the importance of delivering the optimal drug ratio at the target site, since the balance antiproliferative vs. pro-proliferative effects can easily be shifted when the molar ratio between DOX and SIM changes.

## 1. Introduction

A 2020 statistic from the World Health Organization highlighted that colorectal cancer (CRC) is the third most common type of cancer, and the second one based on the number of deaths [1]. The conventional therapy for CRC consists of surgical resection of the tumor, chemotherapy, radiotherapy, or a combination of these [2]. Usually, chemotherapy implies the use of high concentrations of chemotherapeutic drugs with low therapeutic indexes and reduced specificity, which further leads to the appearance of an increased number of adverse reactions with low patient compliance and complementary healthcare support/costs [3]. On the other hand, more and more cancerous cells develop drug resistance to chemotherapy through different mechanisms of action, emphasising the need to explore new therapeutic paths [4,5]. To overcome this issue, combined therapy is considered a preferred option due to the synergistic effect obtained with good results in clinical practice [5] by increasing the survival rate among CRC patients [2,6]. Currently, the recognized combinatorial chemotherapy in CRC is an association of two or three chemotherapeutic active substances selected after examining the beneficial-risk balance and by considering the stage of cancer [6]. However, the systemic administration of two drugs in a free form brings major disadvantages. One of them is that the doses used are administered at their maximum tolerated dose, leading to cumulative toxicity in both tumor and healthy tissues. Additionally, the overall result might not be the expected one, due to different biopharmaceutical properties (biodistribution, metabolization, elimination) of the associated active substances [7,8]. Considering these aspects, it is of critical importance that both active substances reach the target site at the same time and in the required concentration to exert the desired cytotoxic effect [5,8]. Liposomes, of all types of nanoparticles, present the unique ability to form complex nanosystems by incorporating hydrophilic and lipophilic substances [9], thus representing a viable solution for the co-encapsulation of two active substances with different physio-chemical properties.

Doxorubicin (DOX) is amongst the most used chemotherapeutic agents due to its large spectrum of action [10]. Despite this, the drawbacks of DOX therapy (high distribution profile, low therapeutic index, enhanced organ toxicity particularly cardiotoxicity) have inspired researchers to encapsulate it into nanoparticles [11,12]. The first pharmaceutical nanoformulation approved by the drug agencies were liposomes encapsulated with DOX (Doxil^®^). It was evidenced through clinical trials that the use of liposomes in clinical practice may bring several benefits, including a decrease in the number of side effects or an improved therapeutic response due to the targeted action of liposomes at the tumor site [9,13]. In this regard, a phase II clinical study investigated the benefits of ThermoDox^®^, a liposomal formulation with DOX that exhibits thermosensitive properties, in association with thermal ablation in CRC patients with liver metastasis [14]. The results were not yet published, but the use of DOX in CRC therapy was/is the subject of other clinical trials [15,16,17,18], suggesting the potential benefits of this active substance in CRC therapy.

However, the major challenge in CRC therapy is represented by the tumor microenvironment. As a consequence of the anatomical position of CRC tumors, the continuous alterations of the physiological conditions and the constitution of tumors influence the therapeutic response [19]. Recently, an increased number of studies have investigated via in vitro and in vivo experiments various combinatorial chemotherapy regimens with compounds from different classes, i.e., statins, curcuminoids, or isothiocyanate [20,21,22], as means to enhance the cytotoxic effect of conventional chemotherapeutic drugs. Among these classes, statins were the only ones that were tested in clinical trials and provided an improved therapeutic response due to their pleiotropic effects, i.e., antiproliferative, anti-inflammatory, and antioxidant [23,24,25]. Additionally, it was demonstrated that statin therapy has a cardioprotective effect on women with breast cancer who followed a therapy with anthracyclines by reducing the number of heart failure hospitalizations [26]. Among statins, simvastatin (SIM) represents a promising option for a potential association with DOX for two reasons. Firstly, SIM possesses a lipophilic character which was demonstrated to enhance its diffusion in cells, thus exerting a greater cytotoxic effect on cancerous cells [25], and, secondly, it was pointed out that this association enhances DOX cytotoxic effect on numerous cancer cell lines, such as prostate cancer, neuroblastoma, leukaemia, breast cancer, or CRC [20,27,28,29,30].

The successful incorporation of SIM and DOX in liposomes was already achieved in a previous work [20], and the study evidenced that the association of SIM and DOX in liposomes induces a more pronounced inhibitory effect on PC3 prostate cancer cell line and human umbilical vein endothelial cells, compared to free drugs. Despite this, the study has several drawbacks, such as the poor presentation of the selected liposomal formulation and of the release profile of SIM from liposomes. To overcome these drawbacks, the use of the Quality by Design (QbD) concept in the pharmaceutical development of liposomes can be considered a promising and practical option. Tefas et al. applied the QbD concept in the development of DOX and curcumin liposomal co-formulation, highlighting different influences of formulation factors and process parameters on liposomes quality attributes. Moreover, the design space obtained in this study facilitated the achievement of a liposomal formulation in which the two active substances were incorporated at the desired drug ratio in order to obtain the highest inhibitory effect on C26 murine colon cancer. On the other hand, the release study evidenced that the total percentage of drug released can vary depending on the chemical properties of the active substance and the compartment in which the active substance is located in liposomes [30]. The incorporation of two active substances in a liposomal formulation can be a complex and challenging process in relation to preparation technique [31,32]. Starting with the approval of Doxil^®^, several attempts have been made to optimize the active loading of DOX into liposomes [33,34,35], but, when co-encapsulating two active substances, the quality profile of the formulation can be significantly influenced by several variables. To our knowledge, there is only one study that investigated and optimized the passive loading of a statin along with the active loading of DOX into liposomes [36]. The major disadvantage of this study was that the research group focused on optimizing only the encapsulation efficiency of the active substances, without considering the other quality attributes of liposomes, i.e., size, polydispersity index, and drug to drug ratio, which are also critical for an enhanced anticancer effect [36]; therefore, through this study, we aimed to achieve a more in-depth evaluation of the quality profile. The implementation of the QbD concept in drug development is currently a strategy applied in scientific research, as well as in industrial manufacturing. Pharmaceutical formulations developed by QbD are meeting patient needs and predetermined quality conditions [37]. There are currently approved products, such as Gazyva^®^, Gazyvaro, or Januvia^®^, that include in the marketing authorization application elements of this concept [38]. Beyond that, the QbD strategy can also be used in industrial manufacturing to optimize different processes, such as wet granulation or tablet coating, with the aim of reducing the variability between batches [38].

Given these points, this study aimed to investigate the pharmaceutical development of liposomes with long-circulating properties and co-encapsulating SIM and DOX (SIM-DOX-LCL) by implementing the QbD approach. In this regard, the QbD concept, by means of risk assessment tools, design of experiments (DoE), and statistical analysis, was used to “incorporate the previous knowledge” and to interlink the experimental results with the independent variables considered potential critical for the quality attributes of liposomes, with the aim to obtain the co-formulation that meets the quality target profile [37]. The main advantage of QbD is that helps in achieving an extensive knowledge about the preparation process and facilitates the scientific based decisions in the matter of determining the variables that influence the most the quality profile of the formulation/product [37]. Firstly, literature research was performed with the aim to establish the quality target product profile (QTPP) and to define the ranges of liposomes critical quality attributes (CQAs) in order to attain the desired therapeutic performances. Based on these objectives, a wide range of formulation factors and process parameters were analyzed using risk assessment tools for their potential impact on liposomes CQAs. The risk assessment evidenced that three formulation factors (phospholipids (PL), DOX, and SIM concentrations) and two process parameters (incubation time and pH of the ammonium sulphate (AS) solution) were at the highest risk to influence SIM-DOX-LCL CQAs. Secondly, the identified factors were assayed through a screening experimental design, and the experimental results were statistically analyzed to point out the variation of CQAs in relation to formulation factors and process parameters. Lastly, the in vitro performance of SIM-DOX-LCL was followed through determining the release profile of SIM and DOX depending on PL concentration, and through evaluating the cytotoxic profile at different drug ratios on C26 murine colon cancer cells in co-culture with macrophages. To the best of our knowledge, this is the first study that evaluated the preparation process of SIM-DOX-LCL in a systematic manner, providing a detailed understanding of the overall development procedure, and highlighted the link between the quality attributes of liposomes and the anticancer features of the liposomal formulation on C26 murine colon cancer.

## 2. Materials and Methods

### 2.1. Materials

For liposomes preparation, the following were used: doxorubicin hydrochloride (DOX) from Merck KGaA (Darmstadt, Germany), simvastatin (SIM) from Biocon Limited (Bengaluru, India), 1,2-dipalmitoyl-sn-glycero-3-phosphocholine (DPPC) and N–(carbonyl-methoxypolyethylenglycol-2000)-1,2-distearoylsn-glycero-3-phosphoethanolamine (Na-salt; MPEG-2000-DSPE), from Lipoid GmbH (Ludwigshafen, Germany); cholesterol (CHO) from sheep wool, from Merck KGaA (Darmstadt, Germany); sodium chloride and ammonium sulphate (AS), from Chemical Company (Iasi, Romania). For cell cultures, the following were used: C26 murine colon carcinoma cells, from Cell Line Services (Eppelheim, Germany); RPMI 1640 containing L-glutamine, HEPES, antibiotics, from Lonza Group Ltd. (Basel, Switzerland); fetal calf serum, from Merck KGaA (Darmstadt, Germany). All the other solvents and reagents were of analytic grade purity, commercially available.

### 2.2. Methods

#### 2.2.1. Implementation of the QbD Concept

The implementation of the QbD concept in the development of SIM-DOX-LCL was conducted in accordance with international guidelines, namely International Conference on Harmonization (ICH) guideline for pharmaceutical development Q8(R2) and the guidance for industry for liposome drug products approved by the Food and Drug Administration (FDA) [37,39]. The main steps followed in this procedure were: (1) defining the QTPP and identification of the CQAs of SIM-DOX-LCL; (2) identification of material attributes and process parameters that might influence the CQAs using risk assessment tools (Ishikawa diagram and Failure Mode Effects Analysis (FMEA)); (3) development and performance of the DoE; and (4) data analysis.

The QTPP and the target value of CQAs were established based on the existing literature. Additionally, the material attributes and process parameters identified as potential critical for liposomes CQAs were summarised in an Ishikawa diagram. By using FMEA as a second tool for risk analysis, each quality attribute was analyzed from the perspectives of possible effects that might occur if the quality target is not accomplished, along with the potential causes and control methods. For each potential cause identified, a risk priority number (RPN) was calculated, representing the product between severity (S), occurrence (O), and detection (D). RPN value illustrates the potential severity of each effect produced by the identified factor, the probability of failing due to the potential cause and the easiness of detecting the problem. Factors were ranked from 1 (the lowest impact) to 5 (the highest impact). The potential causes that received the highest RPN values were considered potential critical for the quality of SIM-DOX-LCL and, therefore, were studied in a screening DoE.

(1) RPN=S×O×D

The DoE was generated using Modde 12.1 software (Sartorius Stedim Data Analytics AB, Umea, Sweden). The matrix of independent variables was set based on the risk analysis results, and it included PL, SIM, and DOX concentration, the pH of the AS solution, and the incubation time of DOX with long-circulating liposomes encapsulated with SIM (SIM-LCL). The range value of each factor was established with reference to the published literature. The matrix of dependent variables was represented by the CQAs of SIM-DOX-LCL, namely SIM and DOX entrapped concentration, SIM and DOX encapsulation efficiency (EE%), liposomal size before and after the incubation process of SIM-LCL with DOX, and polydispersity index (PdI) before and after incubation process of SIM-LCL with DOX, as well as zeta potential. Data analysis was performed using the statistical mode of the aforementioned software, and the statistical parameters for the analysis of variance (ANOVA) were calculated.

#### 2.2.2. Liposomes Preparation

SIM-DOX-LCL were obtained by active loading of DOX into SIM-LCL, previously prepared by a modified film hydration method [40,41,42,43]. The main steps of the preparation technique are presented hereafter. The lipid components, namely DPPC, MPEG-2000-DSPE, CHO (molar ratio 95:5:10), and SIM, were dissolved in ethanol in a round bottom flask, followed by solvent evaporation under pressure. The obtained film was hydrated with an AS solution (250 mM) adjusted to pH 5.00 or 5.50 in accordance with the DoE. Both steps were performed using a rotavapor at a temperature of 45 °C. The resulting dispersion was downsized using a LiposoFast LF-50 equipment (Avestin Europe GmbH, Mannheim, Germany) and polycarbonate membranes with pore size of 800, 200, and 100 nm. For the establishment of a pH gradient, the liposomal dispersion was dialyzed against saline using a Slide-A-Lyzer cassette with a molecular weight cut-off of 10 kDa (Whatman^TM^, Cytiva, Little Chalfont, Buckinghamshire, UK), for three hours. After that, SIM-LCL were incubated at 60 °C under stirring with a solution of DOX in saline in a volume ratio of 4 to 1 [44], for a predetermined period of time, in accordance with the DoE. Finally, the liposomal dispersion was dialyzed for 24 h at 4 °C using saline.

Long circulating liposomes encapsulated with DOX (DOX-LCL) were prepared following the same procedure, without adding SIM in the lipidsolution.

#### 2.2.3. Characterization of Liposomes

##### Determination of SIM and DOX Entrapped Concentration

Drug entrapped concentration was determined using a high-performance liquid chromatography (HPLC) method with ultraviolet (UV) detection. The method employed a gradient elution, as follows: in the first two minutes, in the composition of the mobile phase, acetonitrile (ACN) increased from 20% ACN and 80% formic acid 0.1% to 60% ACN and 40% formic acid 0.1%, being kept at this level up to six minutes. The absorbance was read at 245 nm.

Liposomes were dissolved in methanol 1:50 (*v*/*v*) and 10 µL of the stock solution were injected into an Agilent 1100 Series HPLC system (Agilent Technologies, Santa Clara, CA, USA) equipped with a Zorbax C18 column (3.5 μm) (Phenomenex, Torrance, CA, USA). The retention time (R_t_) was 0.95 ± 0.2 min for DOX and 4.5 ± 0.2 min for SIM. The quantification method was validated, and the statistical parameters can be found in the Supplementary file (Tables S1 and S2).

Drug EE% was calculated as a ratio between drug entrapped concentration and total concentration of drug used, using the next formula:

(2) $$\mathrm{EE}\%=\frac{Drug\;entrapped\;concentration}{Total\;drug\;concentration}\text{× }100$$

##### Determination of Liposomal Size and PdI

Liposomal size, PdI and zeta potential were determined using a Zetasizer Nano ZS90 analyzer (Malvern Instruments Co., Malvern, UK). The technique used for the determination of liposomal size and PdI was dynamic light scattering with a scattering angle of 90°, and for zeta potential was laser Doppler electrophoresis. For the analysis, the dispersion was diluted 1:100 (*v*/*v*) in water. All the measurements were performed in triplicate at room temperature.

##### Determination of Liposomal Morphology using Transmission Electron Microscopy (TEM) Analysis

TEM analysis was performed on SIM-LCL and SIM-DOX-LCL prepared at different PL and drug ratios. For this analysis, negative staining method with ammonium molybdate was used, but, prior to this procedure, liposomes were firstly treated with osmium tetroxide 4% in order to obtain a better contrasting image [45,46]. The technique implied the dilution of liposomal suspension (200µL) in PBS (1 to 7 *v*/*v*) and the addition of 1.25 µL osmium tetroxide 4%. The obtained suspension was kept for 1 h at 4 °C. After this period, the liposomal dilution was centrifugated for 5 min at 12,000× *g*, and the sediment was resuspended in 200 µL ultrapure water. From the final suspension, a volume of 5 µL was deposited on carbon-coated copper grids for 2 to 5 min, after which the excess of the sample was removed using filter paper. The sample was then treated with 5% ammonium molybdate and 1% trehalose and left to dry at room temperature in a desiccator. After 24 h, the samples were analyzed using a Hitachi HT7700 electron microscope (Hitachi Ltd., Tokyo, Japan) equipped with a camera.

##### In Vitro Release Study

For the in vitro release study, a modified dialysis cassette method was used [30,47] as follows: two milliliters of liposomal suspension were introduced in a Slide-A-Lyzer cassette with a molecular weight cut-off of 10 kDa (Whatman^TM^, Cytiva, Little Chalfont, Buckinghamshire, UK), which was further immersed in 100 mL of phosphate buffer solution (PBS) pH 5.00 containing 30% ethanol. Ethanol was added to ensure sink conditions for SIM release [47]. The release study was performed at a temperature of 37 ± 0.5 °C under continuous stirring at 100 rpm. At regular intervals, one ml of sample was withdrawn and it was replaced with an equal volume of fresh medium to maintain release conditions. All the samples were analyzed through the HPLC method described formerly. The experiments were performed in triplicate and the results were expressed as mean ± standard deviation.

Two formulations with similar drug entrapped concentrations Table 1 but different PL concentrations, 44 and 77 mM, were evaluated in terms of SIM and DOX release profiles.

To determine the similarity in the release profiles of each active substance between the two formulations, two statistical parameters, namely the difference factor (f_1_) and the similarity factor (f_2_), were calculated using DDSolver add-in software [48].

#### 2.2.4. Cell Co-Culture

All procedures involving mice were performed according to the EU Directive 2010/63/EU and to the national regulations. The study is reported in accordance with ARRIVE guidelines and approved by the Babes-Bolyai University Ethics Committee (Cluj-Napoca, Romania; Approval no. 12917/10.07.2019). Bone marrow was collected from 6–8-week-old Balb/c male mice from the femoral bone, as it was previously described by Zhang et al. [49]. The femurs were collected and deposited in 70% ethanol, being washed for 1 min with the same solution. After that, the epiphyses of the femur were removed and with a syringe, the bone marrow was flushed in culture medium, and passed through a cell strainer to obtain a uniform single-cell suspension. Cell suspension was centrifugated for 10 min at 400× *g* and 4 °C. Supernatant was withdrawn and cell pellet was resuspended in DMEM medium containing 10% inactivated fetal bovine serum and 20 ng/mL M-CSF for 7 days to allow macrophage differentiation from marrow progenitors. Macrophages were maintained at 37 °C and a 5% CO_2_ humidified atmosphere, until co-cultured with colon carcinoma cells.

C26 murine colon carcinoma cells (Cell Lines Service GmbH, Eppelheim, Germany) were cultured in RPMI-1640 medium containing 10% inactivated fetal bovine serum and were maintained at 37 °C in a 5% CO_2_ humidified atmosphere.

Co-cultures were obtained by seeding macrophages and cancer cells in a ratio of 1 to 4, as previously described [50,51].

#### 2.2.5. Cell Proliferation Assay

Co-cultures were exposed for 48 h to different treatments, i.e., DOX or/and SIM in free form or in liposomes. Tested concentrations varied between 0.007 and 10 µM for DOX as a free form, respectively, and 0.045 and 20.76 µM for SIM. DOX in combination with SIM (0.93 or 3 µM) was varied between 0.015 and 8 µM as a free form, while, in liposomes, between 0.007 and 5 µM as single therapy or in combination with SIM. The molar ratio between SIM and DOX in liposomes was either 4 to 1 or 12 to 1. The cytotoxicity of the treatment was determined using ELISA BrdU-colorimetric immunoassay (Merck Applied Science, Mannheim, Germany) according to the manufacturer instructions [52]. All the experiments were performed in triplicate, and the results are expressed as % of inhibition of cell proliferation.

All statistical analyses were performed by using GraphPad Prism version 6 for Windows (GraphPad Software Inc., La Jolla, CA, USA). The IC_50_ values for all experimental treatments were determined by non-linear regression of the sigmoidal dose-response curves. To compare the effects of different DOX-based treatments on the proliferation of co-cultured cells, a two-way analysis of variance (ANOVA) with Tukey correction was used. Significance was considered at values of *p* < 0.05 (ns, *p* > 0.05; *, *p* < 0.05; **, *p* < 0.01; ***, *p* < 0.001; ****, *p* < 0.0001).

## 3. Results and Discussions

### 3.1. Setting the QTPP and the CQAs

The experience gained from the development of Doxil^®^ (the first pharmaceutical product with liposomes approved by a drug agency) evidenced that the therapeutic activity of liposomes in cancer treatment is dependent on the quality profile of the formulation [13]; therefore, the QTPP of SIM-DOX-LCL included a range of features which should ensure the antiproliferative effect of the formulation in cancer treatment. These features were set based on literature review. The most relevant characteristics are summarized in Table 2 and were aimed to ensure the surface characteristics of liposomes and the drug load as a means to obtain a prolonged circulation time and a prolonged drug release profile, so that the formulation reaches the tumor site and delivers the desired drug concentration, while the off-target side effects are minimised [13].

### 3.2. Risk Analysis

**Figure 1:**
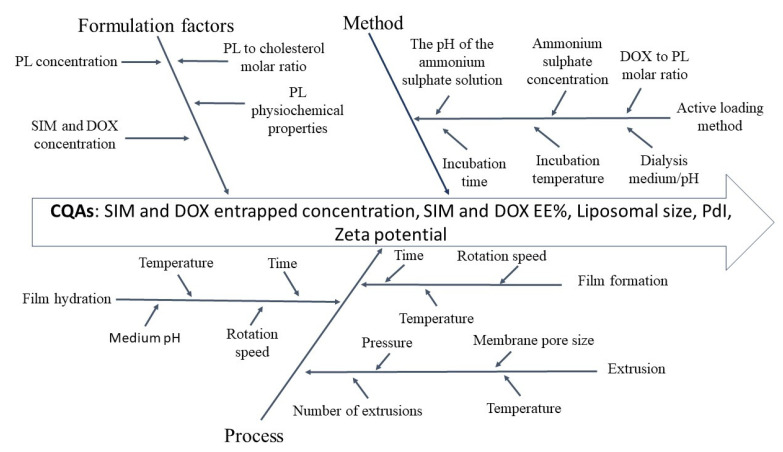
Ishikawa diagram summarizing the material attributes and process parameters with a potential impact on SIM-DOX-LCL CQAs.

Ishikawa diagram Figure 1 was used as a risk assessment tool to summarize the material attributes and process parameters that might present a critical impact on SIM-DOX-LCL CQAs. Given the increased number of identified factors, an additional risk analysis method was employed, namely FMEA. Using FMEA, each CQA, i.e., drug entrapped concentration, EE%, liposomal size, PdI, and zeta potential, was evaluated from the perspectives of failure effects, potential causes, and control methods. The results of the analysis are presented in a condensed form in Table 3 and are widely discussed in the following paragraphs.

According to the FDA guideline for liposomes development, a liposomal system consists of an active substance, lipids, and other components, a major role in liposomes performance being ascribed to the physio-chemical properties of lipids and their concentration [39]. Different studies evidenced that physical properties of lipids, i.e., transition temperature (T_m_), hydrophobicity or saturation level, influence the encapsulation of DOX and improved result being obtained in the case of saturated lipids [40]. Other advantage of the use of saturated lipids is attributed to their increased T_m_, therefore preventing the leakage of encapsulated drug during blood circulation [9]. On the other hand, it was observed that the exclusive use of saturated lipids leads to a decreased EE% for DOX, and the incorporation of cholesterol in the lipid bilayer increases it [40,53]. This behavior can be explained by the fact that cholesterol reduces the hydrophobic character of the lipid bilayer, enabling the incorporation of DOX into the aqueous core [40]. Considering that we aimed to encapsulate two active substances in liposomes, namely DOX in the aqueous core and SIM in the lipid bilayer, the impact that cholesterol levels have on SIM encapsulation was also evaluated. In this regard, Porfire et al. noted that elevated concentrations of cholesterol led to a decrease in SIM encapsulation [43], perhaps due to the role of cholesterol in decreasing the hydrophobic character of lipid bilayer [53]. Given the considerations listed above, DPPC was used as the main phospholipid due to its saturation level, T_m_ (41 °C) and the fact that a high EE% for DOX can be achieved using this lipid [40,54]. Moreover, the molar ratio between PL and cholesterol was established at 10 to 1, to achieve a high EE% for SIM, as well [43].

The incorporation of hydrophilic active substances into liposomes is usually performed using thin film hydration method but, in most cases, leads to a reduced EE% [53]. In the case of DOX, it is unfeasible to incorporate a significant quantity of DOX into nano liposomes, required to achieve a favorable therapeutic outcome in cancer treatment [13]. As a result, an active loading method using AS was developed [13], that led to an EE% exceeding 90% for pharmaceutical products such as Doxil^®^ and Caelyx^®^ [55]. Starting from this point, numerous development studies were performed to determine the optimal conditions for DOX encapsulation into liposomes. So far, it was concluded that ammonium salts in comparison with sodium salts provide a higher EE%, and a salt concentration of 250 mM is needed for the same purpose [34]. On the other hand, the incubation temperature and time of DOX with the liposomes need to be optimized depending on liposomes composition [56]. However, previous results evidenced that an increased EE% was obtained when a temperature of 60 °C was used for the incubation of DOX with DPPC and cholesterol containing liposomes [34,40]. Our preliminary results (data not presented) also evidenced that an increase in incubation temperature from 45 °C to 60 °C led to an increase in DOX EE% with 20%; thus, 60 °C was used as the optimal temperature for the incubation process of SIM-LCL with DOX.

According to the literature, in the active loading method using the AS gradient, a pH difference between liposomes interior and exterior is not mandatory [33,53], but a decrease in pH value of the AS solution is suggested to interfere with this method, preventing the ammonium ion dissociation, while an increase of the pH leads to a favorable effect [33,57]. Considering this and based on working conditions used by others [58,59], two values of pH for the AS solution, namely 5 and 5.5, were assessed to determine their influence on SIM-DOX-LCL CQAs. Moreover, Bolotin et al. found that a critical parameter in AS active loading method is the efficient removal of AS from external medium in order to create a pH gradient. They observed that, when the external liposomal medium was substituted with saline (pH 5.5), the liposomal internal pH dropped from 7.45 to 3.6 [60]. In our preliminary studies (data not presented), liposomes were dialyzed against PBS pH 7.4 and saline for 24 h or 3 h, and the results did not evidence a significant difference in DOX EE%. However, zeta potential exhibited more negative values when liposomes were dialyzed against saline compared to PBS, result which is in accordance with other reports [61]. Considering this, the optimal conditions for the establishment of pH gradient have been set to 3 h dialysis against saline.

The limitations of active loading method are related to drug to lipid molar ratio, a value greater than 0.95 not being recommended since it leads to a decreased EE% and may, as well, compromise liposomes integrity as a result of expanded DOX-sulphate crystals [56]. Taking this into consideration, the concentrations of DOX have been set to avoid the aforementioned effect irrespective of the PL concentration.

The therapeutic activity and in vivo fate of liposomes are conditioned by quality attributes of liposomes, such as size, PdI, as well as by the incorporation of PEGylated lipids in liposomes bilayer [9,13,62]. The most studied mechanism through which liposomes target the tumor site is passive targeting, using the loopholes of tumor vasculature [9]. For this to be accomplished, liposomes smaller than 200 nm are recommended [9], assuring, at the same time, an increased blood circulation time [63]. In light of this, the extrusion process of liposomes was optimized in the preliminary studies by using membranes with a pore size of 200 and 100 nm, and by increasing the number of extrusion cycles until no further decrease in liposomal size or PdI value was observed. The inclusion of PEGylated lipids is aimed to avoid liposomes uptake by reticuloendothelial system macrophages and their fast elimination [5,62], but special attention must be paid to molecular weight of PEG, as well as to PEGylated-lipid concentration, since these material attributes can influence the drug release profile of liposomes [64].

Zeta potential is a CQA that provides information about liposomes stability; values lesser than −30 mV or higher than +30 mV are recommended for a good stability of the suspension, but a minimum value of −20 or +20 mV is also acceptable [65]. Another aspect for which the zeta potential is important is that it can dictate the interaction of nanoparticles with different target cells, the biodistribution, or the drug release [65,66]. Bearing this in mind, different comparative studies were performed, and the conclusions evidenced that negatively charged liposomes are more suitable for intravenous administration, since they present a reduced cytotoxic effect on blood components and an increased blood circulation time [65].

Considering these issues, FMEA analysis has highlighted that five factors might have a potential impact on liposomes CQAs. These included PL, DOX, and SIM concentration, the pH of the AS solution, and the incubation time of SIM-LCL with DOX. According to Table 3, these factors presented the highest RPN value and, consequently, were further studied through a screening DoE. We also assessed PdI value as a potential indicator of the even encapsulation of DOX into liposomes, given that drug crystals of various dimensions can be obtained in function of the formulation variations [67].

### 3.3. DoE

Considering the increased number of identified potential critical factors, a L_18_ fractional factorial (mixed level) experimental design with three center points and 21 experiments was designed and performed, of which three were replicates. All the experimental results achieved after the performance of the DoE are presented in Table 4. The data was fitted through the multiple linear regression (MLR) model and the ANOVA parameters were calculated.

### 3.4. DoE Analysis

#### 3.4.1. The Influence of the Formulation Factors on Drug Entrapped Concentration

The drug entrapped concentration varied between 126.6 and 2066.85 µg/mL for SIM and between 59.64 and 410.57 µg/mL for DOX. The values of statistical parameters showed a good fitting of the data with the proposed models since R^2^ was 0.90 for SIM and 0.94 for DOX, while Q^2^ was 0.73 for SIM and 0.87 for DOX. The independent variables had a great influence on these responses given that the *p*-values of the regression models were smaller than 0.001 for both active substances, and the *p*-value for the lack of fit was 0.49 for SIM, and 0.36 for DOX.

**Figure 2:**
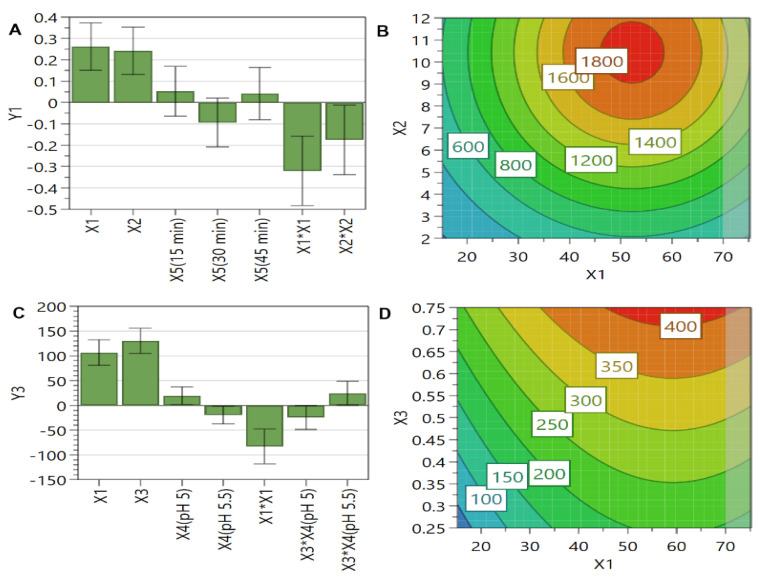
The coefficient plots for SIM (**A**) and DOX (**C**) entrapped concentration showing the significance of formulation factors in respect to these responses. The response contour plots for SIM (**B**) and DOX (**D**) entrapped concentration showing the variance of experimental results in respect to PL concentration (X1), SIM concentration (X2), and DOX concentration (X3). X1: PL concentration (mM); X2: SIM concentration (mM); X3: DOX concentration (mM); X4: the pH of the AS solution; X5: incubation time with DOX (min); Y1: SIM entrapped concentration (µg/mL); Y3: DOX entrapped concentration (µg/mL).

The coefficient plot (Figure 2A,C) reveals that the most critical factors for these responses were PL concentration and SIM or DOX concentration, all three factors presenting a positive influence. In addition, the contour plot, as well as the coefficient plot, revealed the nonlinear manner by which PL concentration influenced these responses. In case of SIM (Figure 2B), the maximum entrapped concentration was achieved at high levels of SIM concentration (>8 mM) and medium levels of PL concentration (between 40 and 60 mM), a further increase of these factors leading to a decrease in SIM entrapped concentration. Regarding DOX entrapped concentration (Figure 2D), a continuous increase of this response was observed when both formulation factors, namely PL concentration and DOX concentration, were enhanced.

The dosing interval of a liposomal formulation/drug product and the patient’s safety are dependent on the drug entrapped concentration in liposomes [68]. Because of this, the Guidance for Industry for liposomes development included the drug entrapped concentration in the list of the CQAs of liposomes to be considered in the preparation/manufacturing process [39]. Yet, an elevated drug concentration in liposomes is desired from the perspective of enhancing patient compliance by increasing the dosing intervals. It is known that by increasing PL concentration, more active substance is embodied in liposomes due to the formation of an increased number of liposomes [53]. However, a contrast regarding the encapsulation behavior of the two active substances could be observed in Figure 2B,D. We assume that the decrease of SIM entrapped concentration with the increase of PL and SIM concentration can be ascribed to the extrusion process and to the interaction of SIM molecules with the lipid bilayer [69,70]. More precisely, at high levels of PL concentration, more SIM molecules are attached to PL, but, since the PL concentration decreases with the number of extrusion cycles and the pore size of the membrane [71], the SIM entrapped concentration will, consequently, decrease. By contrast, DOX entrapped concentration increases constantly with the increase of PL concentration, since this response is dependent on the number of formed liposomal vesicles and PL concentration preserved after the extrusion process. As it was previously mentioned, the active loading of DOX implies the formation of DOX-sulphate crystals inside the aqueous core of liposomes [72]; therefore, the more liposomal vesicles are formed, a higher concentration of DOX can be entrapped. In addition, Alves et al. [73] demonstrated that DOX can form hydrogen bonds with the PL of the lipid bilayer, which can further lead to an increase of DOX entrapped concentration with the increase of PL concentration.

#### 3.4.2. The Influence of the Formulation Factors on EE%

EE% ranged from 2.52 to 70.71% for SIM and from 28.67 to 97.16% for DOX. Statistical analysis showed a good fitting of the data with the proposed models since the values of R^2^ were greater than 0.90, and of Q^2^ were greater than 0.75 for both responses. ANOVA test results indicated that the independent variables presented a significant impact on these responses, considering the *p*-values for the regression models, which were smaller than 0.001 for both active substances, and the *p*-value for lack of fit was 0.67 for SIM, and 0.61 for DOX.

**Figure 3:**
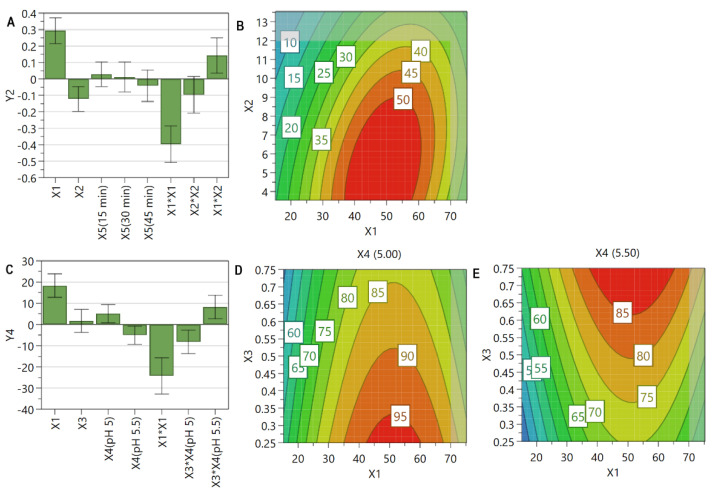
The coefficient plots for SIM (**A**) and DOX (**C**) EE% showing the significance of formulation factors in respect to these responses. The response contour plots for SIM (**B**) and DOX EE% when the pH of the AS solution was 5.00 (**D**) or 5.50 (**E**) showing the variance of experimental results in respect to PL concentration (X1), SIM concentration (X2), DOX concentration (X3), and the pH of the AS solution (X4). X1: PL concentration (mM); X2: SIM concentration (mM); X3: DOX concentration (mM); X4: the pH of the AS solution; X5: incubation time with DOX (min); Y2: SIM EE% (%); Y4: DOX EE% (%).

Figure 3 highlights that PL concentration is a critical material attribute influencing both DOX and SIM EE% (Figure 3A,C). Increasing the levels of both formulation factors, namely PL concentration and SIM or DOX concentration, EE% increased up to a certain point, after which a decrease of its value was observed. Additionally, the coefficient plot of DOX EE% revealed an interaction between the pH of the AS solution and elevated concentrations of DOX, a lower pH presenting a negative influence on this response, while a higher pH an opposite effect. This interaction was also confirmed through the contour plot (Figure 3D,E). At a closer look, it could be noticed that, at increased levels of DOX, the EE% was similar for both values of pH, but a higher EE% was achieved when DOX concentrations were low, and the pH value of AS solution was 5.00 (Figure 3D,E).

Previous studies evidenced that PL and active substance concentration represent critical material attributes in relation to EE%, a presumption that is also supported by our results. Increasing PL concentration resulted in generation of a large number of liposomes that are able to encapsulate more active substance [53]. However, at elevated PL concentrations (>60 mM), a decrease in EE% for both active substances was observed. This might be attributed to the losses during the extrusion process, the liposomal suspension being more viscous at high levels of PL, leading to a hindered extrusion process and increased losses of liposomes components [53]. Additionally, the high number of extrusion cycles and the use of membranes with small pore size have led to the same effect [69]. The differences in DOX EE% at the two values of pH might be explained by the influence of the pH on liposomes bilayer fluidity at different temperatures. In a previous study it was demonstrated that liposomes prepared at acidic pH are the stiffest below T_m_, but, as the temperature raises, they become more fluid compared to liposomes prepared at greater pH [74]. Considering this aspect, we can assume that liposomes prepared at pH 5.00 are more fluid at 60 °C, enabling DOX to diffuse into liposomes to form DOX crystals and, therefore, to achieve a greater EE%. At pH 5.5, the liposomes were stiffer, and the EE% enhanced with DOX concentration considering that more DOX was available for the encapsulation process. On the other hand, the decrease in EE% with the increase of SIM concentration at the same concentration of PL (Figure 3D) can be ascribed to the stiffening effect of SIM on the lipid bilayer [75]. Based on this fact, it is possible that high levels of SIM prevent liposomes deformation and formation of DOX sulphate crystals with larger dimensions, resulting into a decreased efficiency of DOX encapsulation.

#### 3.4.3. The Influence of the Formulation Factors on Liposomal Size

The liposomal size ranged from 108.86 to 135.76 nm and from 105.83 to 132.36 nm, before and after the incubation step, respectively. The values of statistical parameters (R^2^ = 0.97 or 0.90; Q^2^ = 0.94 or 0.75) showed a particularly good fitting of the data with the proposed models. Considering the ANOVA test results, the independent variables proved to have a significant influence on these responses (*p* < 0.001), and the proposed models demonstrated the absence of the lack of fit (*p* = 0.123).

**Figure 4:**
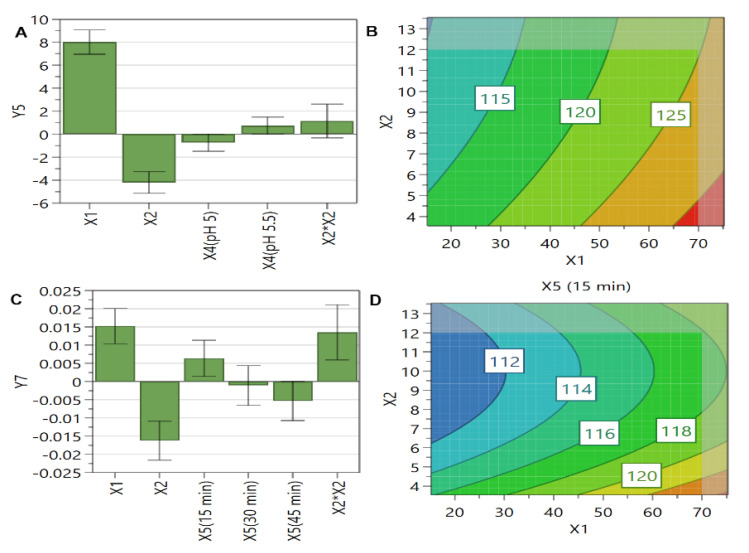
The coefficient plots for liposomal size before the incubation with DOX (**A**) and after the incubation with DOX (**C**) showing the significance of formulation factors in respect to these responses. The response contour plots for liposomal size before the incubation with DOX (**B**) and after the incubation with DOX (**D**) showing the variance of experimental results in respect to PL concentration (X1) and SIM concentration (X2). X1: PL concentration (mM); X2: SIM concentration (mM); X4: the pH of the AS solution; X5: incubation time with DOX (min); Y5: liposomal size before the incubation with DOX (nm); Y7: the liposomal size after the incubation with DOX (nm).

Previous studies have shown that liposomal size is dependent on the selected size reduction method [69,76] and liposomes composition [77]. Considering that the extrusion process was optimized at the beginning of the study in order to obtain a homogeneous dispersion with respect to size, the differences occurred between formulations are the result of the influence of the formulation factors and process parameters. The size of SIM-LCL was shown to be mainly influenced by PL and SIM concentration (Figure 4A). An increase in PL concentration has led to an increase in liposomal size, while an increase in SIM concentration to an opposite effect (Figure 4B). As discussed, the use of increased PL concentration results in a more viscous and stable dispersion that is difficult to extrude and consequently leads to formation of vesicles with increased dimensions [43]. On the other hand, an increase in SIM concentration was observed to lead to smaller vesicles, a result ascribed to SIM, which exhibits a stiffening effect on the lipid bilayer [75]. The hardening effect that SIM exerts on lipid bilayer might also have consequences on the extrusion process, leading to the rupture of the liposomes membrane [78] and, possibly, to the formation of smaller vesicles.

The incubation process of SIM-LCL with DOX proved to have no significant effect on liposomal size (Figure 4C), even though a very slight decrease in the liposomal size was observed after the incubation process (Table 2 and Figure 4D). The lowering effect on vesicle size (Figure 4D) might be attributed to the osmotic imbalance produced by the encapsulation process of DOX, causing a shrinking effect on liposomes when DOX concentration was smaller than 20 mol% [58].

#### 3.4.4. The Influence of the Formulation Factors on PdI

The experimental values of the PdI varied between 0.014 and 0.065 before the incubation process and between 0.022 and 0.091 after the incubation process, showing a good fitting of the data with the proposed model. This affirmation is sustained by the values of statistical parameters, R^2^ and Q^2^, which were 0.87 and 0.69 before the incubation step of SIM-LCL with DOX and 0.90 and 0.82 after this step. The ANOVA test results showed that the independent variables presented a great influence on this response (*p* < 0.001). Moreover, the proposed model did not present a significant lack of fit since the *p*-value was 0.616 and 0.611.

**Figure 5:**
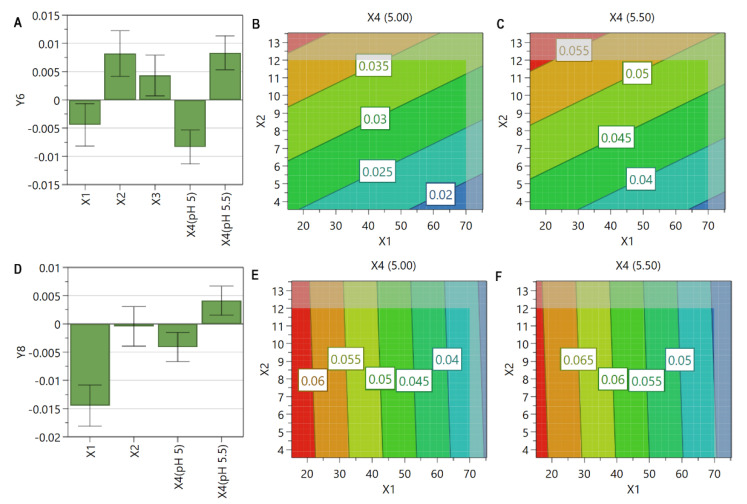
The coefficient plot (**A**) and the response contour plot for PdI before the incubation with DOX when the pH of the AS solution was 5.00 (**B**) or 5.50 (**C**). The coefficient plot (**D**) and the response contour plot for PdI after the incubation with DOX when the pH of the AS solution was 5.00 (**E**) or 5.50 (**F**). The coefficient plots evidence the significance of formulation factors in respect to these responses. The response contour plots evidence the variation of experimental results in respect to PL concentration (X1), SIM concentration (X2), and the pH of the AS solution (X4). X1: PL concentration (mM); X2: SIM concentration (mM); X3: DOX concentration (mM); X4: the pH of the AS solution; Y6: PdI before the incubation with DOX; Y8: PdI after the incubation with DOX.

As it is presented in Figure 5, the formulation factors, namely PL concentration, SIM concentration, and the pH of the AS solution, evidenced a linear influence on PdI values. The contour plot revealed that PL concentration presented a negative influence on this response, while SIM concentration an opposite effect, regardless the pH of the AS solution. It has been pointed out that high concentrations of SIM increase the rigidity of liposomes membrane, therefore influencing the extrusion process and, consequently, the liposomal size and PdI values. This can be sustained by the work of Doskocz et al., who affirmed that “the flow of the liposomes suspension across the extrusion membrane depends predominantly on the lipid bilayer tensile strength and bending rigidity” [78]. However, an increase of PdI value was noted when the pH of the AS solution was 5.50 (Figure 5B,C), a result also sustained by the coefficient plot (Figure 5A). As it was previously presented in Section 3.4.2, liposomes prepared at a lower pH are more fluid at elevated temperatures [74]; therefore, a more homogeneous dispersion was obtained after the extrusion process.

The incubation process of SIM-LCL with DOX had a leveling effect on PdI values in well-defined ranges of PL concentration, regardless of SIM or DOX concentration (Figure 5E,F). It could also be observed that the differences in PdI values between the two values of pH were also reduced by the incubation process (Figure 5B,C,E,F). Furthermore, the negative influence of PL concentration on PdI value was more evident after the incubation process (Figure 5A,D), with lower results being obtained at elevated concentrations of PL.

The variations in DOX crystal dimensions depending on the formulation factors were confirmed through PdI measurements, given the raised values of PdI after the incubation of SIM-LCL with DOX. Achieving similar PdI values irrespective of DOX concentration might indicate that the increased number of extrusion cycles generated liposomes with a restricted internal volume which have limited the width of DOX crystals. On the other hand, increasing PL concentrations caused a decrease in PdI value due to formation of a greater number of vesicles with an increased stability and size for DOX encapsulation.

#### 3.4.5. The Influence of the Formulation Factors on Zeta Potential

For all formulations, zeta potential exhibited negative values in the range of −20.70 mV to −37.93 mV. The statistical parameters showed that the results fitted well with the proposed model since the values of the two statistical parameters, R^2^ and Q^2^, were 0.86 and 0.71. The studied formulation factors had a great impact on this response, considering the *p*-value of the regression model which was smaller than 0.001. The ANOVA test revealed that there is no significant lack of fit of the proposed model since the *p*-value was 0.517.

**Figure 6:**
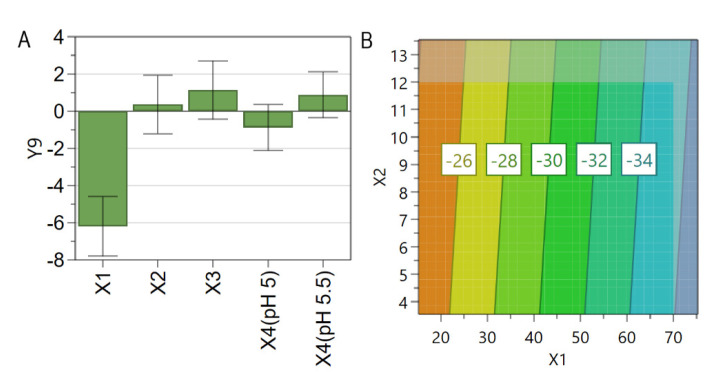
The coefficient plot (**A**) for zeta potential response showing the significance of formulation factors in respect to this response. The response contour plot (**B**) for zeta potential response showing the variance of experimental results in respect to PL concentration (X1) and SIM concentration (X2). X1: PL concentration (mM); X2: SIM concentration (mM); X3: DOX concentration (mM); X4: the pH of the AS solution; Y9: zeta potential (mV).

For zeta potential, the most critical factor proved to be the PL concentration. The negative influence of this factor (Figure 6A) is well represented in the contour plot (Figure 6B), with an increase of PL concentration leading to a decrease in zeta potential value.

It is well known that the variations in zeta potential stem from the DPPC main attribute to own positive and negative charges simultaneously, the prevailing charge depending on the preparation conditions, such as temperature, pH, or ionic strength [79,80]. In a previous work, it was highlighted that phosphate moieties from DPPC can bind anions or cations, thus influencing the results [79]. We assume that, by enhancing PL levels, more chloride anions from liposomes external medium attached to DPPC molecules and determined a decrease of zeta potential values.

### 3.5. TEM Analysis

TEM analysis was performed on two liposomal formulations, namely N3 and N13. Liposomes were analyzed before and after the incubation of SIM-LCL with DOX. These formulations were selected based on the coefficient plots for size and PdI, showing that, at low levels of PL and high levels of SIM, the liposomal size was small, but the PdI value was high, and vice versa. Additionally, both formulations were prepared using the same DOX concentration (0.75 mM), which is the highest concentration used in this study, and could cause alterations in liposomes structure, given that the PdI values increased after the incubation step (Figure 5D–F).

**Figure 7:**
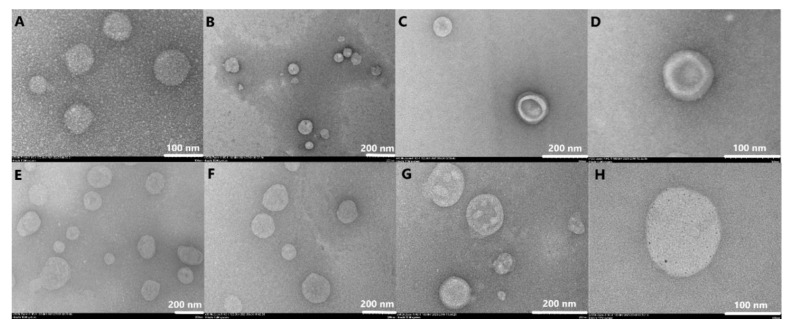
TEM images showing the morphology of N3 and N13 liposomal formulations. N3 before (**A**,**B**) and after (**C**,**D**) the incubation process. N13 before (**E**,**F**) and after (**G**,**H**) the incubation process. The images were taken at different resolutions for a better imaging. The scale bar is represented in the right bottom for each image.

For N3, TEM images (Figure 7A–D) evidenced that liposomes were spherical in shape and uniform in size before, as well as after, the incubation process. Additionally, it was noted that SIM-DOX-LCL were characterized by a deflated liposomal center (Figure 7C,D). As for N3, the images of N13 (Figure 7E–H) evidenced spherical shape of liposomal vesicles, but no visible changes in liposomal structure were noted after the incubation step.

The flattening effect observed for N3 might explain the slight decrease of liposomal hydrodynamic size after the incubation of SIM-LCL with DOX (Figure 4D) and confirm the theory that an osmotic imbalance is created during the incubation process. However, this effect was more pronounced at low levels of PL, since it was not visible for N13 (Figure 7G,H). Different morphological aspects of DOX crystals in relation to drug to lipid ratio, were also reported by Ruiz et al. [81]. They evidenced via cryo-TEM images that increasing the drug to lipid ratio, DOX crystals presented a round shape inside the liposomes. This result might also explain the “doughnut” shape of N3 formulation.

### 3.6. In Vitro Release Study

Risk analysis (Table 3) highlighted that PL concentration might be a critical factor in the release profile of SIM and DOX; therefore, two liposomal formulations with similar drug entrapped concentrations and different PL concentrations were assessed for this study.

The release medium was chosen considering the lipophilic character of SIM [82] and the pH-dependent stability of DOX, as well as the pH of the tumor microenvironment [83]. Prior to the release study, various release conditions were tested as a means to determine the sink conditions for both active substances (data not presented). Results highlighted that PBS pH 5.00 is suitable for DOX release, while an addition of 30% ethanol in the release medium is essential for SIM release, these results being in agreement with the observations of other authors [30,47].

**Figure 8:**
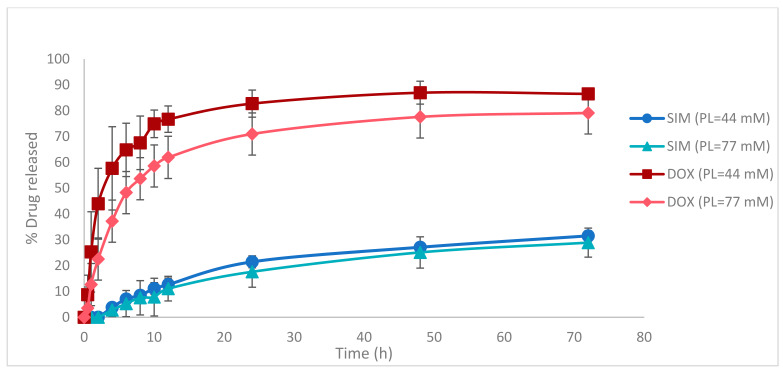
SIM and DOX release profiles from formulations with different PL concentrations. The results are expressed as % of drug released from the total drug entrapped concentration/ ml of liposomes, at different time intervals. The vertical bars at each sampling point represent the standard deviation of three samples.

The release profile evidenced that DOX exhibited a burst release in the first 10 h, followed by a prolonged release up to 72 h for both formulations, while SIM exhibited a prolonged release for the entire duration of the study irrespective of the formulation (Figure 8).

In addition to that, it was proved that PL concentration plays a critical role in the release profile of DOX. Even though both formulations evaluated presented similar release patterns, the total percentage of DOX released was higher (85.44%) when PL concentration was lower compared to the other formulation (79.12%). The f_1_ and f_2_ values were 22.15 and 42.9, respectively, and evidenced that the two formulations are not similar in respect to DOX dissolution profile. On the other hand, the release of SIM was influenced to a lesser extent by PL concentration (31.51% versus 28.92%), a result also sustained by the f_1_ and f_2_ values which were 13.9 and 83.21, and indicated that the evaluated formulations are similar in respect to SIM release profile.

In cancer treatment, the drug release rate from liposomal formulations can be a limiting factor in achieving the therapeutic response, various factors being able to influence it, such as size, liposomal composition, or drug solubility [44,63,84]. For both formulations, DOX exhibited a biphasic release (Figure 8), a burst release in the first 10 h, followed by a sustained release up to 72 h. Previously, it was demonstrated that the solubility of the precipitate in the release medium and the diffusion rate of the active substance through the lipid bilayer determine the feature of the release curve [85,86]. Thus, the acidic pH of the tumor microenvironment determines a twofold increase of DOX leakage from liposomes compared to the physiological pH, as demonstrated by Russel et al. by assessing the leakage rate of DOX sulphate from liposomes in various experimental conditions [86]. Another factor that may have contributed to the burst release of DOX is the presence of ethanol, which leads to an increase of DPPC bilayer surface area [87]; therefore, more molecules of DOX can diffuse through the lipid bilayer. However, the differences between the two formulations in the cumulative DOX released (Figure 8) might be related on the one hand to the influences that SIM exerts on the lipid bilayer, and on the other hand to the PL concentration. Sariisik et al. demonstrated that an increase in SIM mol% leads to a decrease in T_m_ [75], which may favor the release of DOX at low levels of PL. On the other hand, the release rate of hydrophilic active substances can be reduced by increasing PL concentration and liposomal size [88]. In another study, it was reported that the in vitro release of DOX from liposomes is also dependent by the shape of DOX crystals, which are, in turn, influenced by the PL concentration [81]. In view of this, we may conclude that DOX release profile was influenced by both SIM mol% and PL concentration, and a balance between the two factors needs to be achieved in order to prevent drug leakage during blood circulation and to attain the therapeutic outcome.

Regarding SIM release profile, no significant differences were observed between the evaluated formulations, but a large fraction of SIM remained trapped inside the lipid bilayer. The tendency of SIM to form strong interactions with nanoparticles without affecting the cytotoxic effect was also observed in previous works [47,89]. Depending on the lipophilic character of each statin, the interaction with the hydrophobic tail of PL can occur at different levels of depth of the lipidic bilayer, with the lipophilic statins being located more profoundly [90]. Therefore, the diffusion process of SIM is delayed, which consequently leads to a slow release rate from the liposomal formulation.

### 3.7. The Effects of SIM and DOX on the Proliferation of C26 Murine Colon Carcinoma Cells Co-Cultured with Murine Macrophages

Solid tumors are complex structures constituted of various types of cells, such as cancer cells, fibroblasts, endothelial cells, and immune cells, such as macrophages, T and B cells, dendritic cells, etc., which interconnect with each other through different signaling pathways and determine the alteration of physiological conditions, thus forming the tumor microenvironment [91,92]. Among the most prominent immune cells that infiltrate in the tumor, macrophages were demonstrated to play a key role in the progression, invasion and drug resistance of CRC [93]. By co-culturing C26 murine colon carcinoma cells with macrophages, we aimed to simulate the tumor microenvironment of CRC and to target both cell types, using the proposed combination of active substances.

**Figure 9:**
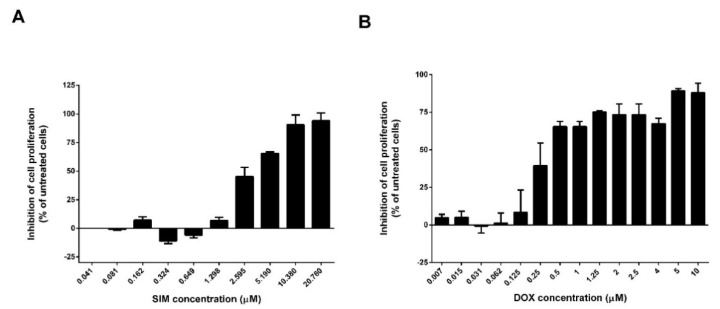
The effects of SIM and DOX on the proliferation of C26 murine colon carcinoma cells co-cultured with murine macrophages. The results show cell proliferation at 48 h after incubation of C26 murine colon carcinoma and murine macrophages, with 0.041–20.76 μM SIM (**A**) or with 0.007–10 μM DOX (**B**), as free forms. Data are presented as the mean ± standard deviation of triplicate measurements. The results are expressed as percentage of inhibition of co-cultured cells proliferation following SIM or DOX treatments, compared to the proliferation of control cells (untreated cells). Significance was considered at values of *p* < 0.05 (ns, *p* > 0.05; * *p* < 0.05; ** *p* < 0.01; *** *p* < 0.001; **** *p* < 0.0001).

**Figure 10:**
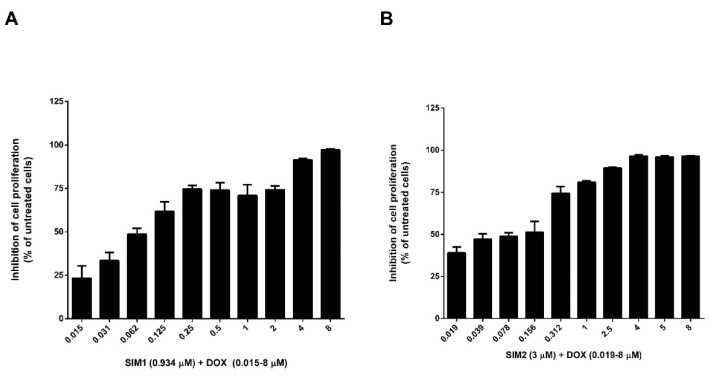
The effects of combined treatment with free SIM and DOX on the proliferation of C26 murine colon carcinoma cells co-cultured with murine macrophages. The results show cell proliferation at 48 h after incubation of C26 murine colon carcinoma cells and murine macrophages with: (**A**) 0.934 μM SIM1 with DOX solutions of variable concentrations (0.015–8 μM DOX) or (**B**), 3 μM SIM2 with DOX solutions of variable concentrations (0.019–8 μM DOX). Data are presented as the mean ± standard deviation of triplicate measurements. The results are expressed as percentage of inhibition of co-cultured cells proliferation following SIM and DOX treatments, compared to the proliferation of control cells (untreated cells). Significance was considered at values of *p* < 0.05 (ns, *p* > 0.05; * *p* < 0.05; ** *p* < 0.01; *** *p* < 0.001; **** *p* < 0.0001).

To investigate whether SIM could potentiate the cytotoxicity of DOX, we determined the effects of free SIM or DOX as monotherapy (Figure 9A,B), as well as of SIM in combination with DOX, on the proliferation of co-cultured cells (Figure 10). SIM in combination with DOX was tested at two levels of variation, one corresponding to SIM IC_50_ value (SIM2) and one corresponding to a lower concentration (SIM1). The two concentrations were assessed considering that SIM may have various effects in a concentration dependent manner. The effects of the treatments were expressed as percentages of the inhibition of cell proliferation compared to the proliferation of the co-cultured cells used as controls. The antiproliferative studies evidenced that SIM exhibited an IC_50_ value of 3 µM (Figure 9A) as a free form, while, for DOX, the IC_50_ value was 0.25 µM (Figure 9B). When the two active substances were combined, the cytotoxic effect of DOX was enhanced for both concentrations of SIM, but, at the IC_50_ value of SIM and variable concentrations of DOX (0.015–5 µM), no evident effect was noted on DOX IC_50_ value (0.27 µM) (Figure 10B). In contrast, a lower concentration of SIM (0.93 µM) determined a decrease of DOX IC_50_ value to 0.06 (4-fold decrease), highlighting a more pronounced inhibitory effect on the co-cultured cells (Figure 10A). When comparing Figure 10A,B, it could be noticed that, at lower concentrations of DOX (0.019–0.078 µM), 3 µM of SIM enhanced the antiproliferative properties of DOX to a greater extent compared to 0.93 µM of SIM. These results suggest that SIM potentiates the cytotoxic effect of DOX; therefore, the incorporation of SIM and DOX in the same liposomal formulation is justified.

**Figure 11:**
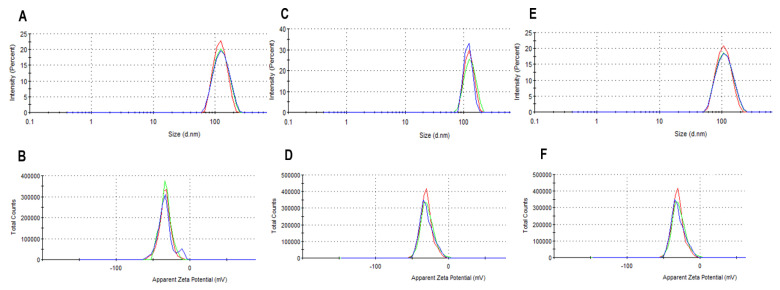
**(A**,**C**,**E**) represent the size distribution of DOX-LCL, SIM1-DOX-LCL, and SIM2-DOX-LCL, respectively. (**B**,**D**,**F**) represent the zeta potential distribution of DOX-LCL, SIM1-DOX-LCL, and SIM2-DOX-LCL, respectively. The measurements were performed in triplicate, and each measurement is represented with a different colour (blue, red, or green).

For the preparation of liposomes, the optimizer function of Modde 12.1 software (Sartorius Stedim Data Analytics AB, Umea°, Sweden) was used to determine the formulations upon which the molar ratios between SIM and DOX can be reached (Table 5). The pH of the AS solution was established to 5.00, while the incubation process of SIM-LCL with DOX to 15 min. The experimental results of DOX-LCL, SIM1-DOX-LCL, and SIM2-DOX-LCL, respectively, are presented in Table 5, while the size and zeta potential distribution is presented in Figure 11.

**Figure 12:**
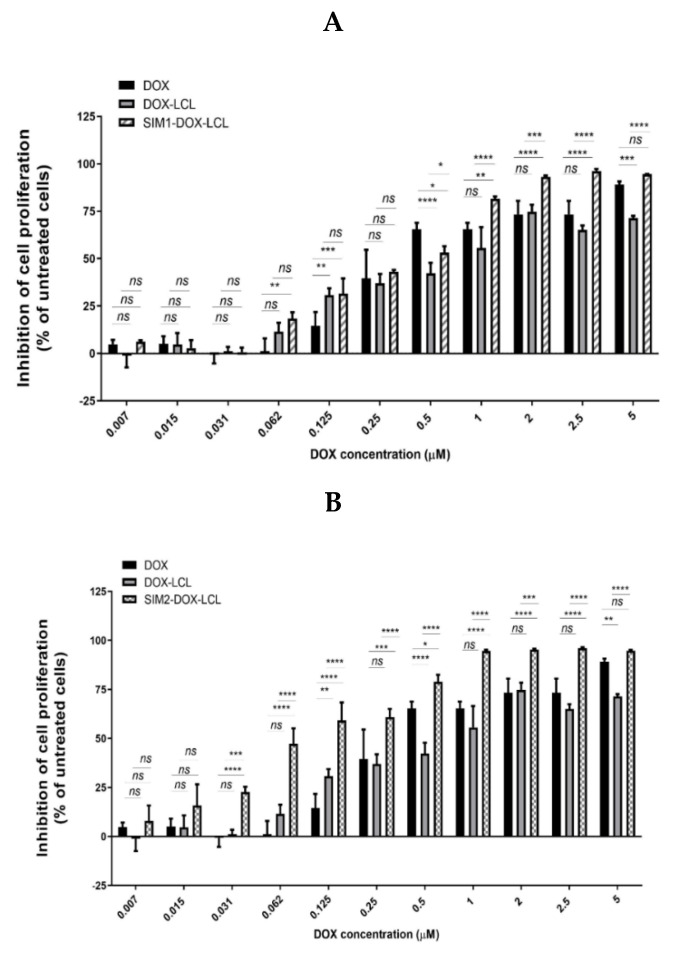
The effects of liposomal co-administration of SIM and DOX on the proliferation of C26 murine colon carcinoma cells co-cultured with murine macrophages. The results show cell proliferation at 48 h after incubation of macrophages and C26 co-cultured cells with free DOX or liposomal DOX. For DOX, three liposomal formulations were tested and statistically compared in terms of the effects on the inhibition of co-cultured cell proliferation: long circulating liposomes encapsulated with DOX (DOX-LCL) (A,B), long circulating liposomes co-encapsulated with SIM (0.93 µM) and DOX (0.23 µM) (SIM1-DOX-LCL (**A**), and long circulating liposomes co-encapsulated with SIM (3 µM) and DOX (0.25 µM) (SIM2-DOX-LCL) (**B**). Data are presented as the mean ± standard deviation of triplicate measurements. The results are expressed as percentage of inhibition of co-cultured cells proliferation following free or liposomal DOX treatments, compared to the proliferation of control cells (untreated cells). Significance was considered at values of *p* < 0.05 (ns, *p* > 0.05; * *p* < 0.05; ** *p* < 0.01; *** *p* < 0.001; **** *p* < 0.0001).

As it is shown in Figure 12, the encapsulation of DOX into liposomes did not improve the aforementioned result (DOX-LCL IC_50_= 0.27 µM). Despite that, the co-encapsulation of SIM and DOX in a molar ratio of 12 to 1 highlighted a reduction of DOX IC_50_ value to 0.08 µM (Table 6), while a ratio of 4 to 1 caused an increase of DOX IC_50_ to 0.37 µM.

The antiproliferative properties of two active substances in combination is highly dependent on their combination ratio due to different therapeutic effects that can be obtained, i.e., antiproliferative or pro-proliferative [94]. In our case, we observed that the combination of the two active substances (as free forms) at their IC_50_ value (SIM to DOX molar ratio of 12 to 1) did not cause any additional beneficial effect compared to monotherapy, but the association of a low dose of SIM with DOX at its IC_50_ value (SIM to DOX molar ratio of 4 to 1) led to a more evident inhibitory effect. This result might be attributed to SIM which, depending on its concentration, might inhibit or promote the proliferation of cells in co-culture, via its modulatory effects on reactive oxygen species (ROS), with key role in cancer cell proliferation [95]. It must be noted that the encapsulation of DOX into liposomes did not improve the antiproliferative effect of DOX compared to its free form, result which might be attributed to liposomal components. In this regard, Horowitz et al. reported that the use of PL with high T_m_ can lead to an increase of IC_50_ value for DOX [96]. On the other hand, we assume that the drug release profile of SIM and DOX played a key role in the antiproliferative properties of the co-formulation. As it was already presented, a higher mol% of SIM promotes DOX release, and, as a result, a more pronounced inhibitory effect was obtained when SIM and DOX were co-encapsulated in a molar ratio of 12 to 1. In summary, the increased release rate of DOX from the co-formulation combined with the antiproliferative properties of SIM have led to an effective antiproliferative effect on C26 murine colon cancer cell line in co-culture with macrophages, but a shift in the results can be obtained depending on the liposomes composition, drug release rate, and drug molar ratio.

## 4. Conclusions

This paper provides evidence that the implementation of the QbD concept in the pharmaceutical development of liposomes is practical and beneficial, since numerous quality attributes of a liposomal nanostructure used for tumor targeting have to be very well controlled to achieve the desired therapeutic outcome. A key factor in this study was the use of risk assessment tools to facilitate the identification and the classification of formulation factors by the level of risk to impact on product quality, which consequently contributed to narrowing the number of assessed variables. Moreover, their evaluation through a screening study, by using a broad level of variation for each formulation factor, provided an insight of the manner in which the formulation factors influence the quality profile of SIM-DOX-LCL. The antiproliferative activity of the formulation against colorectal cancer cells was shown to be dependent on the molar ratio between the two active substances and on their release rate. By using the mathematical models developed in this study, it has been possible to design a liposomal co-formulation with SIM and DOX that was demonstrated to be more noxious on C26 murine colon cancer cells co-cultured with macrophages, when compared with the free forms.

However, additional studies are required to identify the optimal formulation of SIM-DOX-LCL, as well as to characterize it in terms of stability and therapeutic efficacy using different in vitro techniques and in vivo studies, respectively.

## Figures and Tables

**Table 1:** The composition of the formulations used for in vitro drug release studies.

PL (mM)	44	77
DOX (µg/mL)	176.86 ± 25.39	194.9 ± 66.43
SIM (µg/mL)	1267.42 ± 121.96	1552.44 ± 76.13

**Table 2:** The summary of QTPP and CQAs of SIM-DOX-LCL.

Quality Target Product Profile		Target	Justification
Formulation	Liposomes	-	To deliver simultaneously SIM and DOX at the tumor site
Route of administration	Intravenous	-	To increase the bioavailability of both active substances at the tumor site
Quality attributes	Pegylated liposomes	-	To ensure prolonged blood circulation time for liposomes
	Zeta potential	≤−30 mV	Values higher than +30 mV or lower than −30 mV are required for a good colloidal stability of the formulation
	EE%	50–100%	Reduced production losses
	Drug entrapped concentrations	DOX > 120 µg/mL	Increased dosing intervals
		SIM > 1200 µg/mL	
	SIM to DOX molar ratio	12 to 1	Securing the right ratio between the two active substances in liposomal formulation is essential to achieve the desired cytotoxic effect at the tumor site
	Spherical shape	-	Active loading of DOX into liposomes may lead to a deformation of liposomes shape and membrane disruption
	Size	50–200 nm	Enhanced permeability retention effect is dependent on the liposomal size, values smaller than 200 nm being indicated; Liposomes smaller than 50 nm are quickly eliminated by the spleen and through kidney filtration
	PdI	<0.2	A monodisperse liposomal suspension is critical for predictable in vivo behavior
	Prolonged in vitrorelease profile	-	Required in order to increase the dosing interval and to prevent off-site drug release

**Table 3:** Failure mode effects analysis.

Parameter	Failure Mode	Failure Effects	Potential Causes	Control Methods	S	D	O	RPN
Drug entrapped concentration and EE%	-Reduced drug entrapped concentration; -Reduced EE%	-Drug losses; -Increased production costs; -Frequent dosing required; -Enhanced administered doses; -Reduced patient compliance; -Diminished therapeutic effect	PL physiochemical properties	Use of PL with an increased T_m_	3	1	5	15
			PL concentration	Identification of an appropriate concentration range	5	4	5	100
			SIM concentration		5	4	5	100
			DOX concentration		5	4	5	100
			PL to cholesterol molar ratio		5	1	1	5
			DOX loading method	Use of active loading method	5	1	1	5
			Type of sulphate salt	Use of AS salt	5	1	3	15
			Sulphate salt concentration	Use of a 250 mM salt concentration	3	1	3	9
			The pH of the hydration medium	Optimization of the pH of the AS solution	3	4	5	60
			Establishing a pH gradient	Optimization of liposomes purification step	1	1	1	1
			DOX to PL molar ratio	Use of a DOX to PL molar ratio smaller than 0.95	4	1	2	8
			Incubation time	Optimization of incubation time of SIM-LCL with DOX	5	4	5	100
			Incubation temperature	Performing the incubation step at 60 °C	4	1	1	4
Size and PdI	-Multilamellar vesicles; -Polydisperse suspension; -Increased liposomal size	-Reduced internal liposomal volume for DOX encapsulation; -Diminished therapeutic effect; -Fast elimination through reticuloendothelial system; -Reduced colloidal stability	Size reduction method	Application of extrusion process	1	1	1	1
				Optimizing the number of extrusion cycles	5	1	3	15
				Use of membranes with a reduced pore size	4	1	4	16
Prolonged blood circulation time	Rapid removal from blood circulation	-Frequent dosing required; -Diminished therapeutic effect	Formulation of conventional liposomes	Use of pegylated PL	5	1	4	20
Prolonged release profile	Fast drug release rate	-Diminished therapeutic effect; -Frequent dosing required; -Off-site drug release; -Increased number of side effects; -Reduced patient compliance; -Increased health care costs	Use of reduced PL concentration	Optimization of PL concentration	4	4	5	80
			SIM and DOX concentration	Optimization of drug concentration	2	2	5	20
			Use of PL with a reduced T_m_	Use of PL with an increased T_m_	3	1	4	12
Zeta potential	Values in the range of −20 mV to +20 mV	-Reduced colloidal stability; -Interactions with blood constituents and rapid removal from blood circulation; -Vesicle agglomeration; -Sedimentation	PL concentration	Optimization of PL concentration	5	4	5	100
			Ionic strength of the liposomes external medium;	Use of NaCl for dialysis	3	2	2	12
			Type of PL	Use of charged PL	3	3	2	18

S: severity; D: detectability; O: occurrence; RPN: risk priority number; T_m_: phase transition temperature.

**Table 4:** Fractional factorial experimental design and results.

Exp Name	X1	X2	X3	X4	X5	Y1	Y2	Y3	Y4	Y5	Y6	Y7	Y8	Y9
N1	11	2	0.25	5.00	15	157.67	18.77	59.64	28.67	118.46	0.023	110.33	0.028	−22.56
N2	11	7	0.50	5.00	30	297.51	10.20	123.95	42.59	111.86	0.060	106.76	0.091	−20.70
N3	11	12	0.75	5.00	45	126.60	2.52	173.84	38.14	108.86	0.047	105.83	0.066	−25.16
N4	44	2	0.25	5.00	30	364.58	44.05	156.66	91.45	123.33	0.014	122.00	0.036	−34.66
N5	44	7	0.50	5.00	45	1129.08	38.66	325.19	84.20	118.06	0.030	110.16	0.056	−31.20
N6	44	12	0.75	5.00	15	1809.98	35.80	378.42	75.07	117.93	0.025	115.16	0.051	−36.46
N7	77	2	0.50	5.00	15	224.54	27.03	285.83	74.00	132.93	0.050	125.53	0.039	−35.53
N8	77	7	0.75	5.00	30	1130.26	38.61	439.40	87.16	122.50	0.031	117.10	0.060	−34.46
N9	77	12	0.25	5.00	45	1384.17	27.61	202.12	97.16	125.16	0.020	116.80	0.034	−37.00
N10	11	2	0.75	5.50	45	232.02	25.66	189.37	41.55	117.26	0.046	115.30	0.075	−21.13
N11	11	7	0.25	5.50	15	185.80	6.31	61.30	29.47	114.56	0.040	110.36	0.079	−37.93
N12	11	12	0.50	5.50	30	238.31	4.74	167.75	57.64	116.03	0.057	110.70	0.068	−28.50
N13	44	2	0.50	5.50	45	336.65	39.76	275.85	88.19	128.20	0.065	126.00	0.056	−30.93
N14	44	7	0.75	5.50	15	1335.59	45.73	410.57	88.31	120.60	0.046	115.33	0.062	−25.70
N15	44	12	0.25	5.50	30	1200.86	23.97	66.84	39.88	116.86	0.045	109.43	0.059	−27.60
N16	77	2	0.75	5.50	30	244.61	29.22	401.70	79.68	135.76	0.029	132.36	0.040	−31.96
N17	77	7	0.25	5.50	45	1130.51	37.73	111.75	53.72	121.06	0.045	116.03	0.052	−35.53
N18	77	12	0.50	5.50	15	1361.95	27.09	251.59	65.14	120.53	0.052	115.50	0.022	−33.34
N19	44	7	0.50	5.00	15	1969.29	67.21	228.17	78.40	120.86	0.028	114.36	0.054	−29.03
N20	44	7	0.50	5.00	15	2066.85	70.71	274.19	94.21	120.43	0.021	115.76	0.050	−34.63
N21	44	7	0.50	5.00	15	1392.23	47.50	253.12	86.97	119.83	0.045	115.40	0.045	−26.70

X1: PL concentration (mM); X2: SIM concentration (mM); X3: DOX concentration (mM); X4: the pH of the AS solution; X5: incubation time (min); Y1: SIM entrapped concentration (µg/mL); Y2: SIM EE% (%); Y3: DOX entrapped concentration (µg/mL); Y4: DOX EE% (%); Y5: liposomal size before incubation with DOX (nm); Y6: PdI before incubation with DOX; Y7: liposomal size after incubation with DOX (nm); Y8: PdI after incubation with DOX; Y9: zeta potential (mV).

**Table 5:** The composition and the quality attributes of liposomes used in the proliferation assay.

Liposomal Formulation	X1	X2	X3	Y1	Y2	Y3	Y4	Y7	Y8	Y9
DOX-LCL	44	-	0.5	-	-	165.47	56.08	129.4	0.055	−28.7
SIM1-DOX-LCL	44	2	0.25	260.74	35.59	89.90	62.33	119.8	0.050	−28.3
SIM2-DOX-LCL	44	12	0.5	998.49	19.92	115.37	37.27	104.3	0.071	−27

X1: PL concentration (mM); X2: SIM concentration (mM); X3: DOX concentration (mM); Y1: SIM entrapped concentration (µg/mL); Y2: SIM EE% (%); Y3: DOX entrapped concentration (µg/mL); Y4: DOX EE% (%); Y7: liposomal size after incubation with DOX (nm); Y8: PdI after incubation with DOX; Y9: Zeta potential (mV).

**Table 6:** Summary of IC_50_ of SIM and DOX, administered as free or liposomal form.

Pharmaceutical Formulation	Tested Drug Concentration	IC_50_ (μM)
SIM solution	0.041–20.7 μM	3 ± 0.560
DOX solution	0.007–10 μM	0.25 ± 0.047
SIM1 + DOX (variable)	0.934 μM SIM + (0.015–8 μM) DOX	0.06 ± 0.036
SIM2 + DOX (variable)	3 μM SIM+ (0.019–8 μM) DOX	0.27 ± 0.114
DOX-LCL	0.007–5 μM DOX	0.27 ± 0.127
SIM1-DOX-LCL	0.934 μM SIM + (0.007–5) μM DOX	0.37 ± 0.086
SIM2-DOX-LCL	3 μM SIM + (0.007–5) μM DOX	0.08 ± 0.044

## Data Availability

Not applicable.

## Supplementary Materials

The following are available online at https://www.mdpi.com/article/10.3390/pharmaceutics13101526/s1, Table S1. Linear regression parameters for simultaneous quantification of SIM and DOX. Table S2. Evaluation of the accuracy for the simultaneous quantification of SIM and DOX.
