# Supplementary material for: A Screening Study for the Development of Simvastatin-Doxorubicin Liposomes, a Co-Formulation with Future Perspectives in Colon Cancer Therapy
Source: Pharmaceutics. 2021 Sep 22;13(10):1526. doi: 10.3390/pharmaceutics13101526 (PMC8537800; doi:10.3390/pharmaceutics13101526)
Supplement: Supplementary file 1 [file pharmaceutics-13-01526-s001.zip › pharmaceutics-1318370-supplementary.pdf]

pharmaceutics

Supplementary Materials: A Screening Study for the Development of Simvastatin-Doxorubicin Liposomes, a Co-Formulation with Future Perspectives in Colon Cancer Therapy

Cristina Ioana Barbălată, Alina Silvia Porfire *, Alina Sesarman, Valentin-Florian Rauca, Manuela Banciu, Dana Muntean, Rareș Știufiuc, Alin Moldovan, Cristian Moldovan and Ioan Tomuță

Prior to the validation of HPLC method, preliminary experiments (data not presented) were performed, in which the ratios between the two mobile phases were varied with the aim to identify the suitable percentage ratio between acetonitrile (ACN) and formic acid 0.1% (v/v) for the simultaneous quantification of doxorubicin (DOX) and simvastatin (SIM). During the experiments, the peak area as well as the aspect of the peak were analyzed. Results evidenced that a gradient elution was mandatory from the perspective of physiochemical properties of active substances, DOX is a hydrophilic active substance while SIM is a lipophilic one, and time analysis. The best results were obtained when the organic phase was increased from 20% to 40%, while the aqueous phase was decreased from 80% to 60% in the first two minutes. The validation of the HPLC analysis technique was performed in three different days with fresh prepared solutions. Table S1 presents the linear regression parameters for both active substances, while Table S2 shows the tested concentrations for each active substance, and the mean recovery ±standard deviation. The correlation coefficient values for both active substances (>0.98) indicate that the selected concentrations are in the linear range. The mean recovery for all concentrations indicates that the HPLC developed method is accurate.

Table S1. Linear regression parameters for simultaneous quantification of SIM and DOX.

Parameter	SIM	DOX
Retention time (min)	4.5 ± 0.2	0.95 ± 0.2
Specificity	No additional peaks were detected	
Range of linearity (µg/ml)	13-65	1.4–7
Correlation coefficient (R^2)	0.996	0.985
Slope	25.42	8.85
Intercept	104.23	−2.83

Table S2. Evaluation of the accuracy for the simultaneous quantification of SIM and DOX.

Active substance	Concentration (µg/ml)	Mean recovery (%)	RSD (%)
SIM	13	97.70 ± 8.23	8.25
	26	101.03 ± 6.15	6.16
	39	100.64 ± 4.31	4.31
	52	99.83 ± 4.64	4.65
	65	99.81 ± 3.92	3.92
DOX	1.4	95.88 ± 5.44	5.47
	2.8	101.41 ± 8.07	8.10
	4.2	101.05 ± 9.28	9.31
	5.6	100.39 ± 7.49	7.52
	7	99.37 ± 2.57	2.26

RSD = Relative standard deviation.
